# miR-335-laden B Cell-Derived Extracellular Vesicles Promote SOX4-Dependent Apoptosis in Human Multiple Myeloma Cells

**DOI:** 10.3390/jpm11121240

**Published:** 2021-11-23

**Authors:** Elisabetta Lombardi, Gonzalo Almanza, Kinga Kowal, Marco Valvasori, Francesco Agostini, Carla Vicinanza, Francesco Da Ros, Cristina Durante, Miriam Marangon, Mariagrazia Michieli, Maurizio Rupolo, Mario Mazzucato, Maurizio Zanetti

**Affiliations:** 1 Stem Cell Unit, Department of Research and Advance Cancer Diagnostic, Centro di Riferimento Oncologico di Aviano (CRO), IRCCS, 33081 Aviano, Italy; kinga.kowal95@gmail.com (K.K.); marco.valvasori@cro.it (M.V.); fagostini@cro.it (F.A.); carla.vicinanza@cro.it (C.V.); francesco.daros@cro.it (F.D.R.); cdurante@cro.it (C.D.); miriam.marangon@cro.it (M.M.); mmazzucato@cro.it (M.M.); 2 The Laboratory of Immunology, Department of Medicine and Moores Cancer Center, University of California San Diego, 9500 Gilman Drive, La Jolla, CA 2093-0815, USA; galmanza@ucsd.edu; 3 Department of Life Sciences, University of Trieste, 34151 Trieste, Italy; 4 Oncohematology and Cell Therapy Unit, Department of Medical Oncology, Centro di Riferimento Oncologico di Aviano (CRO), IRCCS, 33081 Aviano, Italy; mmichieli@cro.it (M.M.); mrupolo@cro.it (M.R.)

**Keywords:** miR-335, SOX4, multiple myeloma (MM), exosomes, gene therapy

## Abstract

Multiple myeloma (MM) is characterized by the accumulation of malignant plasma cells in the bone marrow. Despite novel therapies, MM still remains an incurable cancer and new strategies are needed. Increased expression of the transcription factor Sex-determining region Y-related high-mobility-group box transcription factor 4 (*SOX4*) has been correlated with tumor development and progression through a variety of distinct processes, including inhibition of apoptosis, increased cell invasion and metastasis, and induction and maintenance of cancer-initiating cells. The role of *SOX4* in MM is largely unknown. Since *SOX4* is a known target of miR-335, we used miR-335 to assess whether *SOX4* modulation could promote apoptosis in MM cells. Using an MM cell model we show that miR-335 acts both on *SOX4*-related genes (AKT, PI3K) and hypoxia-inducible factor 1-alpha (Hif1-α). In addition, we show miR-335-laden extracellular vesicles induced in B cells (iEVs) are also effective in targeting *SOX4*, causing apoptosis. Collectively, we propose that miR-335-laden iEVs could be developed as a novel form of gene therapy in MM.

## 1. Introduction

The annual worldwide incidence of multiple myeloma (MM) is estimated to be 6–7/100,000 and accounts for 1% of all cancer. MM is characterized by the accumulation of malignant plasma cells in the bone marrow microenvironment (BMM). Plasma cells are able to subvert the finely regulated balance between osteoblasts and osteoclasts in bone marrow management to create a self-comfort zone [1]. The interplay between malignant PCs and BMM affects MM progression through paracrine growth circuits while PC adhesion to BM stromal cells is crucial for chemo-resistance [2]. Clinically, MM is characterized, in most cases, by the production of monoclonal immunoglobulins, anemia, hypercalcemia, renal impairment, and lytic bone lesions. MM has complex biology with a multistep process and progressive chromosomal changes, genetic and epigenetic events occurring during the disease course [2,3,4]. Despite the introduction of novel therapies, MM remains an incurable cancer and new strategies are urgently needed.

An important group of transcription factors involved in tumorigenesis and cancer is the sex-determining region Y (SRY)-related high-mobility group (HMG) box (*SOX*). Their name is due to their crucial role in embryogenesis where they control different aspects of tissue development [5]. These characteristics are maintained even in post-natal life so that *SOX*s are engaged in many developmental processes regulating both the maintenance of stem cells and terminal differentiation of a wide variety of cell types [6].

*SOX* genes are also involved in cancer cell proliferation, cell survival, inhibition of apoptosis, and tumor progression through the induction of an epithelial-to-mesenchymal transition (ETM) and metastasis [6]. Increased *SOX4* expression is correlated with poor clinical outcomes in gastric cancer [7], hepatocarcinoma [8], bladder cancer [9], and prostate cancer [10]. In glioblastoma reduced expression of *SOX4* abrogates tumorigenic activity [10] and in breast cancer, *SOX4* overexpression positively correlates with tumor grade and promotes metastasis [11].

It is well known that *SOX4* plays a crucial role in hematopoiesis driving B cell maturation: it is highly expressed in the early stages and its level decreases until basal in B lymphocytes [12]. However, the role of SOX4 in MM cells is poorly understood. Because of its transcriptional activation and repressive roles, SOX4 is expected to regulate a number of genes implicated in cellular development, differentiation, and survival as CD56 [12]. In MM, CD56 expression correlates inversely with bone marrow infiltration and with the number of circulating tumor cells, and higher levels of CD56 are also associated with lytic bone lesions [13]. In addition, *SOX4* expression can be induced by inflammatory cytokines as TGFβ factors, typically present in the tumor microenvironment [14].

Micro RNAs (miRNAs) are short RNA molecules 19 to 25 nucleotides in size that regulate post-transcriptional silencing of the target gene [15]. Due to its short sequence, a single miRNA can bind different mRNAs and subsequently modulate more than one cellular pathway in a direct or indirect way. miR-335 targets *SOX4* mRNA and its expression is downregulated in solid tumors that overexpress *SOX4.* This gene is particularly involved in epithelial developed cancers. In carcinomas, the downregulation of miR-335 is directly correlated with tumor progression and secondary lesion development in hepato-cellular carcinoma, gallbladder carcinoma, breast cancer, and prostate cancer [16,17]. In carcinomas, miR-335 can regulate the sensitivity of tumor cells to anticancer drugs and modifies the metabolic pathway in stromal cells.

Reasoning that the bone marrow microenvironment in MM is crucial for the therapeutic response and clinical outcome, we sought changes in cell viability in MM cells after treatment with a plasmid DNA coding for miR-335-laden extracellular vesicles induced in B cells (iEVs) [18].

## 2. Materials and Methods

### 2.1. B Cell Purification

B cells were obtained by 60 mL buffy coat from healthy donors in compliance with Agreement n.225/CSR, Italian State-Region Conference, 13 December 2018. Cells were isolated by Ficoll-PaqueTM PLUS (density 1.077 g/mL-GE Healthcare Bio-Sciences AB SE-75184 Uppsala, Sweden) followed by immuno-sorting with CD19 Miltenyi beads according to manufacturer instructions (CD19 MicroBeads human, Prod.n. 130-050-301- Miltenyi Biotec GmbH, Germany). After incubation, the mixture of cells and beads was loaded on a column insert in a high magnetic force device from which B lymphocytes (CD19^+^) were eluted and washed. The purity of cells was verified by flow cytometry using an FITC-conjugated monoclonal antibody to CD20 (Clone 2H7, BD Pharmingen™, BD Bioscience, Stockholm, Sweden).

### 2.2. Cells Culture

B cells obtained from Ficoll purification were seeded in RPMI with 10% exosome free fetal bovine serum (FBS) supplemented with 1 mM Na pyruvate, 1% Non-essential amino acids, 1% penicillin/streptomycin (Sigma-Aldrich, St. Louis, MO, USA). U266B1 cells (ATCC^®^ TIB-196™, Manassas, VA, USA) were grown in suspension in RPMI with 10% fetal bovine serum (FBS) with or without the addition of TGFβ-1 (Sigma-Aldrich). Cells were allowed to recover in a T25 flask upright at 37 °C with 5% CO_2_.

### 2.3. Plasmid Construct and Cells Transfection

A miRNA construct containing miR-335 was synthesized with the unique SgfI/XhoI ends by Integrated DNA Technologies (IDT, Coralville, IA, USA). The construct was cloned into the pCMVmir expression vector (Origene, Rockville, MD, USA) by digestion with SgfI and XhoI, and subsequent ligation [18].

B cells and MM cells (10^6^) were transfected with 3 μg of pCMV-miR-335 plasmid using FuGene HD at the 1:6 ratio DNA/transfection reagent (Cat # E2311, Promega Corporation, WI). An empty vector was used as a negative control. Cells were allowed to recover in a T25 flask upright at 37 °C with 5% CO_2_ for 72 h. The viability of U266B1 cells was evaluated by 7AAD (Cat # A1310, Thermo Fisher Scientific, Waltham, MA, USA) on an LSRFortessa™ cell analyzer (BD Bioscience, Stockholm, Sweden). Untransfected cells treated with the transfection agent (sham) and cells transfected with an empty vector were used as controls. The cut-off to discriminate alive from dead cells was set using sham as a reference.

### 2.4. miR-335 Analysis

Total RNA extraction was performed using Trizol RNA Isolation Reagents (Thermo Fisher Scientific, Carlsbad, CA, USA), according to the datasheet instructions, both in whole cells (LBs and U266B1 cells) and in iEVs. The concentration and purity of RNA were determined by analysis on a NanoDrop spectrophotometer (Thermo Fisher Scientific, Massachusetts, MA, USA). cDNA was generated using the iScript™ cDNA Synthesis Kit BioRad (Cat # 1708890, BioRad, CA, USA) according to the manufacturer’s instructions. The reaction was continued as previously described [18].

### 2.5. IEV Isolation and Quantification

One milliliter of culture supernatant from B cells was collected 24 and 72 h post-transfection and incubated with 0.5 mL of Total Exosome Isolation solution (Thermo Fisher Scientific, Waltham, MA, USA) for 1 h at room temperature. The iEV-containing mixture was spun at 16,000 rpm for 1 h at 4 °C. The iEV pellet was resuspended in 100 µL of PBS at room temperature and stored in 1.5 mL Eppendorf tubes at −20 °C until use. The iEVs quantification was estimated as protein concentration using the Bradford test for NanoDrop Technology (Thermo Fisher Scientific). iEVs were numbered by the method of Burkova et al. [19] assuming the iEV mass as 1000 kDa and converting this mass in µg to yield 1 exosome equal to 1660 × 10^−12^ µg. Based on this calculation, we converted the concentration (mg/mL) obtained by NanoDrop quantification in particle number.

### 2.6. B Cell-Derived IEVS and U266B1 Cells

Twenty-four hours after transfection, iEVs were collected from the medium of untreated, sham-, and miR-335-treated B cells. U266B1 cells (2 × 10^5^) were seeded in each well of a 48-well plate and 4 × 10^5^ iEVs/cell were added to the medium. Cytotoxicity was evaluated after 24 and 48 h by the Annexin V (Annexin V, Alexa Fluor™ 555 conjugate–Cat #A35108, Thermo Fisher Scientific, Waltham, MA, USA) assay both using fluorescence microscopy and FACS analysis. Fluorescence imaging assay was performed using Nikon N300 epifluorescence microscope equipped with CFI S Plan Fluor ELWD 40XC (MRH08430) and was collected by NIS-Element software. The quantification of miR-335-positive iEVs was obtained using five independent acquisition data. ImageJ (Fiji Is Just ImageJ, MA, USA) analysis of frames collected at three different time points (0, 24, and 48 h) were analyzed statistically as the mean ± SD, and cell mortality was determined by Annexin V staining.

After iEV treatment, U266B1 cells were analyzed for the positivity to Annexin V also using LSRFortessa™ cell analyzer (BD Bioscience, Stockholm, Sweden). Each experimental condition (Sham, empty plasmid (EP), and miR-335-transfected cells) was analyzed for cell viability in order to perform statistics. We considered the number of dead cells detected in sham-transfected cells as physiological. To estimate the effect of transfection, we normalized the number of dead cells in EP and miR-335 treated cells by subtracting the values of sham-transfected cells.

### 2.7. RT-qPCR

RNA of three different experiments were isolated from U266B1 and B cells using the same procedures previously described. RT-qPCR was performed using the Master Mix and the probe of IDT according to the data sheets. In particular, a 20 µL reaction was made using 100 ng of each sample in a BioRad CFX-96 thermocycler. Target gene expressions were normalized to HPRP1 and analyzed using the -ΔΔCt relative quantification method. Results were expressed as fold change relative to the vehicle-treated control cells, whose gene expression profile did not differ from untreated cells. Validated 6-FAM-labeled human *SOX4* (Hs.PT.58.24974948.g), AKT1 (Hs.PT.58.26215470), PI3KCA (Hs.PT.58.584779), PTEN (Hs.PT.58.4416071), HIF1-A (Hs.PT.58.534274), TGFB1 (Hs.PT.58.39813975) and HPRP1 (Hs.PT.58v.45621572) PrimeTime^®^ Predesigned qPCR Assays (IDT) were used to analyze tumoral cell cDNAs. qPCR data were analyzed and statistical analysis was performed using software.

### 2.8. Western Blot Analysis

U266B1 cells (2 × 10^6^) were lysed in NP40 lysis buffer supplemented with metallo-protease and protease inhibitor cocktails (Sigma-Aldrich, St. Louis, MO, USA). After a Bradford quantification, 40 µg of the whole protein pool was loaded into a 4–20% mini protean TGX gel (Bio-Rad, Hercules, CA 94547, USA). The gel was run at 100 volts for 75 min and then transferred to 0.2 µm PVDF membrane using a Trans-Blot Turbo device (BioRad, Hercules, CA 94547, USA). The Western blotting protocol was followed for antibody detection using specific monoclonal antibodies against different proteins. The following primary antibodies all from OriGene (Austin, TX, USA) were used: mouse monoclonal antibody to human *SOX4* (Cat # TA324704), AKT1 (Cat # TA504230), PI3KCA (Cat # AM06736PU-N), HIF1-A (Cat # AM06608SU-N), and rabbit monoclonal antibody to PTEN (Cat # AP15248PU-N), pAKT1 (Cat # TA313266), TGFβ1 (Cat # TA506585). Bound antibodies were revealed with Clarity Western ECL substrate (Cat # 1705061, BioRad, Hercules, CA 94547, USA). A ChemiDoc™ Imaging Systems (BioRad, Hercules, CA 94547, USA) was used as an imaging system and band quantification was performed using the ImageLab software.

### 2.9. Statistical Analysis

Results are derived from at least three independent experimental tests. All statistical tests were performed using a 2-tailed *t*-test.

## 3. Results

### 3.1. Close Correlation between miR-335 and SOX4 in MM

**Figure 1:**
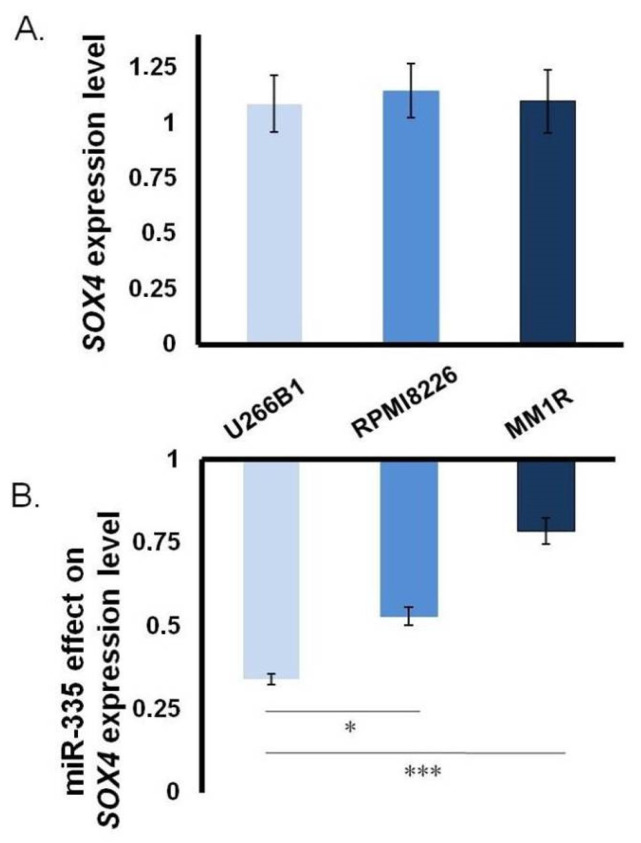
Analysis of *SOX4* RNA expression in MM cells. (**A**) *SOX4* expression level in MM cell lines evaluated by RT-qPCR. The HPRP1 gene was used as housekeeping gene. (**B**) The effect of 72 h transfection with pCMV-miR-335 on different MM cells lines on *SOX4* RNA levels evaluated by RT-qPCR. (*p* < 0.05 = *; *p* < 0.0005 = *** evaluated by *t*-test).

*SOX4* consensus normalized expression value (NX) and *SOX4* protein transcripts per million (pTPM)—described by The Human Protein Atlas—are seven to eight times higher in cancer cells originating from the B line compared to the B lymphocytes [19]. Based on this observation, we investigated the involvement of *SOX4* in MM metabolism by analyzing its mRNA levels in three different MM cell lines: U266B1, RPMI8226, and MM1R. *SOX4* expression was comparable in all the cell lines analyzed (Figure 1A) and was higher compared to the *SOX4* level in B cells (Supplementary Material Figure S1). Next, we checked the ability of miR-335 to downregulate *SOX4* expression. Transfection with pCMV-miR-335 [18] caused a drop in *SOX4* mRNA levels in all three cell lines with U266B1 showing the highest reduction (Figure 1B). In light of this result, we decided to use the U266B1 cell line as a model to study the role of miR-335 and *SOX4* in modulating MM cell behavior.

**Figure 2:**
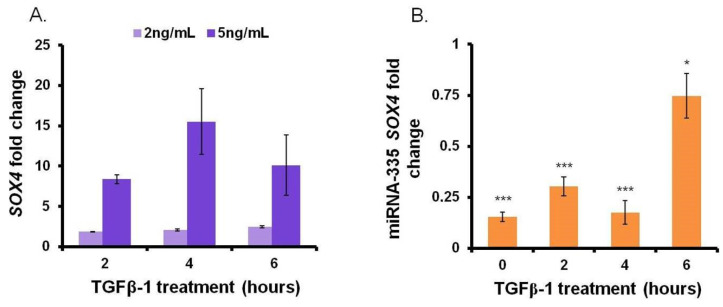
miR-335 regulates *SOX4* mRNA level in U266B1 cells overexpressing *SOX4*. U266B1 cells were transfected with the pCMV-miR-335 plasmid and treated with 2 ng/mL or 5 ng/mL of TGFβ-1 72 h post-transfection. Cells were collected at 0, 2, 4, and 6 h post-TGFβ-1 treatment and analyzed by RT-qPCR. *SOX4* mRNA expression levels were normalized with the HPRP1 housekeeping gene. (**A**) Dose-dependent effect of TGFβ-1 treatment on *SOX4* expression. Fold changes are relative to *SOX4* expression in untreated cells. (**B**) Analysis of *SOX4* mRNA levels in cells transfected with miR-335 and treated with TGFβ-1 (5 ng/mL) for 2, 4, and 6 h, respectively. Fold changes are relative to *SOX4* expression in sham-transfected cells. (*p* < 0.05 = *; *p* < 0.0005 = *** evaluated by *t*-test).

To confirm the specificity of miR-335 as a direct regulator of *SOX4* expression, we first enforced the upregulation of *SOX4* using transforming growth factor-beta 1 (TGFβ-1) according to Dong, M. and Blobe, G. C. [14]. We reasoned that this would mimic natural conditions since both multiple myeloma cells and bone marrow stromal cells (BMSCs) secrete high levels of TGFβ-1 [11]. We observed a dose-dependent correlation between TGFβ-1 and *SOX4* mRNA levels. Two- and ten-fold induction of *SOX4* expression were observed by adding TGFβ-1 at 2 ng/mL or 5 ng/mL concentration, respectively (Figure 2A). Next, we assessed the effect of miR-335 on *SOX4* mRNA level in the presence of TGFβ-1. Cells were transfected with pCMV-miR-355 plasmid and treated with 5 ng/mL of TGFβ-1 at 72 h post-transfection, a window of time required for its expression. Sham-transfected cells were used as a control. As expected, TGFβ-1-induced overexpression of *SOX4* was abrogated by miR-335 (Figure 2B). Collectively, these results confirm the specificity of miR-335 as a regulator of *SOX4* RNA level in MM cells.

### 3.2. Direct Effect of miR-335 Effect on Tumor Cells

**Figure 3:**
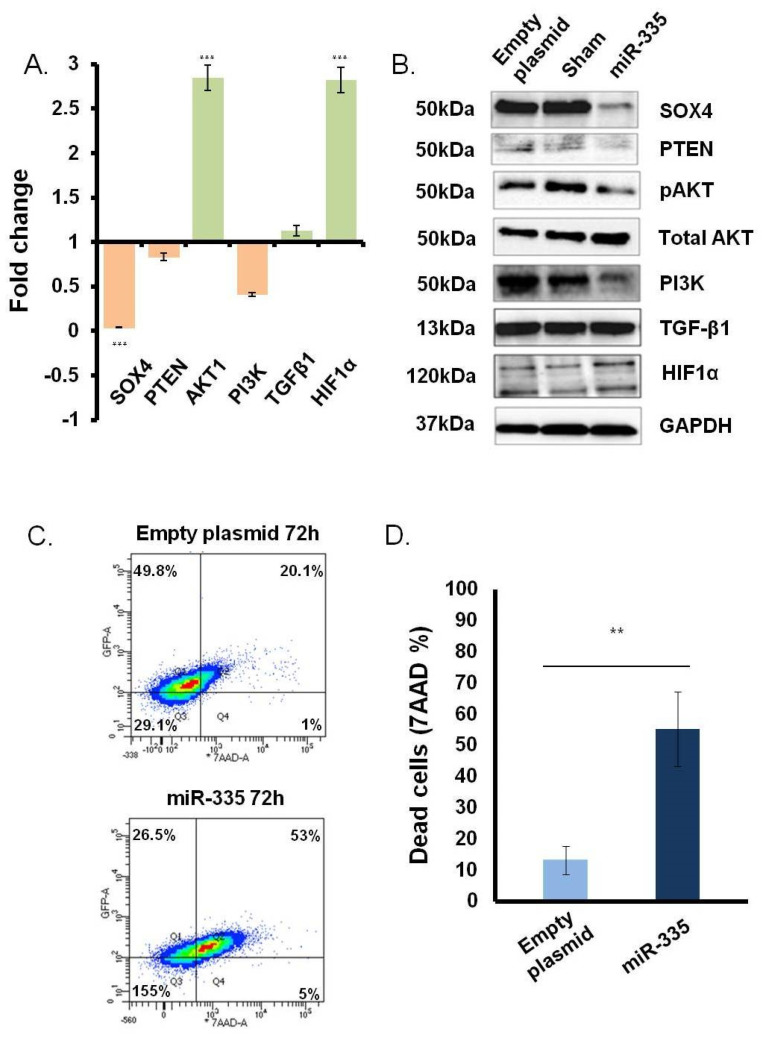
Effect of miR-335 transfection on U266B1 cells. *SOX4* and *SOX4* downstream genes and gene products were investigated at 72 h. (**A**) RT-qPCR assay. The mRNA modulation (green indicates the up-modulation and orange the down) was generated by normalizing miR-335 gene expression with sham-transfected cells. Statistical analysis was performed between three different control and treated cell values (*p* < 0.0005 = *** evaluated by *t*-test). (**B**) Western blot. Empty plasmid transfected cells, sham-, and miR-335-transfected cells were tested for *SOX4*, AKT/pAKT (Ser473), PI3K, PTEN, TGF-β, and HIF1-α. (**C**) Example of flow cytometry 7AAD analysis of U266B1 cells treated as indicated in panel B. (**D**) Cell mortality (7AAD positivity) served in miR-335- vs. empty plasmid-transfected cells. Values were normalized with sham-transfected cells. Data are representative of three independent experiments. (*p* < 0.005 = ** evaluated by *t*-test).

To evaluate the ability of miR-335 to directly regulate *SOX4* in the MM U266B1 cells without the confounding effects of TGFβ-1 we transfected cells with the pCMV-miR-335 plasmid. In pilot experiments, we observed that miR-335 was efficiently expressed starting 48 h post-transfection (data not shown). Accordingly, studies on *SOX4* gene expression, protein levels, and cell viability were consistently performed at 72 h post-transfection. Sham-transfected and cells transfected with empty vector were used as control. Modulation of *SOX4* and other key genes involved in the *SOX4* pathway was assessed by qPCR and Western blotting. As expected, there was a marked decrease (~90%) in *SOX4* gene expression compared to the levels in control cells. A marked reduction was also observed by Western blotting (Figure 3B). In addition to *SOX4*, we also found a marked down-regulation of PI3K/AKT phosphorylation and PTEN (Figure 3A,B). It is well known that the hypoxia-inducible factor-1α (HIF1α) suppresses MM growth and inhibits angiogenesis, acting as a key hypoxia-responsive gene [20]. The miR-335 expression also enhanced both HIF1α gene transcription and protein expression starting from 24 h (data not shown).

To assess cell viability after miR-335 treatment, we performed a 7AAD assay by flow cytometry. A clear and marked increase (from 15% to 50%) in the 7AAD zone in miR-335-transfected cells suggested that overexpression of miR-335 decreased cell viability (Figure 3C). This effect was significant (*p* < 0.005) and consistent across three independent experiments (Figure 3D). Collectively, these results suggest that *SOX4* is a key regulator of MM growth and survival as observed previously in carcinoma models [6,8,21].

### 3.3. B Cells as Efficient Bioreactor for iEVs Production

**Figure 4:**
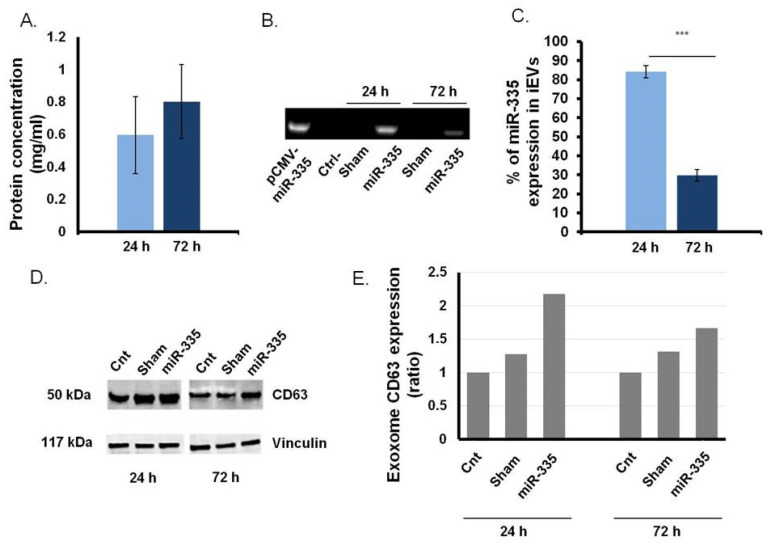
Analysis of B cell-derived iEVs. (**A**) iEVs production estimate as protein concentration at 24 and 72 h. B cells were stimulated, or not, using FuGene HD transfection agent. iEVs were collected from the B cell growth medium using a separation kit as indicated in the Material and Methods. Protein concentration determined by subtracting the values in unstimulated cells from the stimulated ones. (**B**) Detection of miR-335 in iEVs from transfected B cells vs. vesicles from untransfected B cells. (**C**) Densitometry quantitation (percentage over vesicles from untransfected cells as control) of miR-335 in iEVs from transfected cells (*p* < 0.0005 = *** by *t*-test). (**D**) CD63 ex-pressZion on iEVs by Western blot analysis. (**E**) Western blot quantification of CD63. Vinculin, a housekeeping gene, was used for normalization (see Materials and Methods).

We used an established protocol to generate miR-335-laden iEVs [18]. As expected, transfected B cells generated twice as many iEVs as control cells (data not shown). iEV production was relatively constant at 24 and 72 h (Figure 4A). Only iEVs released by B cells transfected with the miR-335 coding plasmid were positive for miR-335 by qPCR (Figure 4B). miR-335 content in iEVs decreased progressively from the 24 h (85%) to the 72 h (28%) time points (Figure 4C). By Western blotting, iEVs expressed higher (×1.5) levels of CD63, a canonical exosome marker, than EVs from sham-transfected B cells. This effect was prominent at 24 h (Figure 4D) but disappeared at 72 h post-transfection (Figure 4E), suggesting, perhaps, that miR-335 enhances exosome biogenesis.

### 3.4. IEVs Effect on U266B1 MM Cells

First, we verified the internalization efficiency of iEVs by U266B1 cells. We used the method of Burkova et al. [19] to estimate the EVs yield and calculate the final iEV concentration to add to MM cells. Twenty-four hours post-transfection, iEVs (4 × 10^5^ iEVs/cell) were added to the medium containing exosome-free 10% FBS. Because miRNA half-life in mammalian cells is short [18], we set the experimental conditions at 24 and 48 h, respectively.

**Figure 5:**
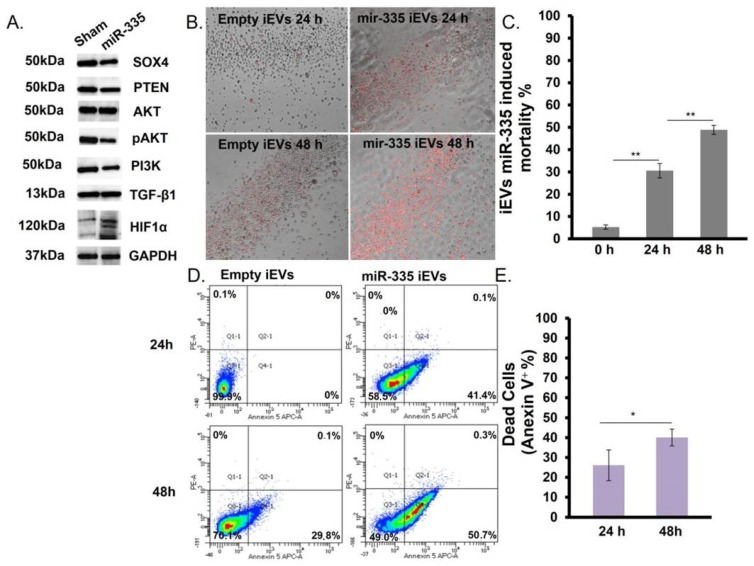
miR-335-laden iEVs modulate *SOX4* and drive apoptosis in U266B1 cells. (**A**) Western blot analysis of protein levels in U266B1 cells after 24 h co-culture with iEVs. (**B**) Fluorescence microscopy of Annexin V (red)-positive cells: empty iEVs and miR-335-laden iEVs were incubated for 24 or 48 h with U266B1 cells at the following ratio (4 × 10^5^ iEVs/cell). The image represents the merge of bright field and fluorescence (Tric). (**C**) Quantification of the effect (cell death) of miR-335-laden iEVs on U266B1 cells. (*p* < 0.005 = ** evaluated by *t*-test). (**D**) Example of flow cytometry analysis of cell death (Annexin V positivity). (**E**) Quantitation of cell death in U266B1 cells treated with miR-335-laden iEVs. Results refer to the mean ± SD of three independent experiments. Statistical analysis was performed using normalized values, and by setting the threshold on empty vesicle-treated cells. (*p* < 0.05 = * evaluated by *t*-test).

Proteomic analysis of *SOX4* and its pathway in U266B1 cells (Figure 5A) showed the downregulation of *SOX4* (~45%) but also of protein downstream of the *SOX4* such as PTEN, AKT, and PI3K (Figure 5A), consistent with what was observed after direct transfection of MM cells (Figure 3A,B). Notably, treatment with miR-335-laden iEVs also induced a marked upregulation of HIF1α.

The effect of iEVs treatment on U266B1 MM cells was also investigated by fluorescence microscopy and flow cytometry. The cell viability was estimated in terms of phosphatidylserine increase (Annexin-V positivity). After 24 h we found a 3-fold higher amount of phosphatidylserine compared to baseline (time zero). An increase in death was observed after 48 h with a total cell death > 50% (Figure 5B,C). To confirm this result, samples were analyzed by flow cytometry and it was found that miR-335-laden iEVs were more efficient at causing cell death than empty iEVs (45% vs. 25%) (Figure 5D) The number of apoptotic cells increased by 20% in the following 24 h (Figure 5E). Altogether, these results suggest iEV-335 is an efficient vehicle to deliver miRNAs for therapeutic purposes.

## 4. Discussion

The treatment landscape for MM has changed dramatically over the last 10 years. Today, standard clinical protocols for MM include high dose chemotherapy with hematopoietic stem cell transplantation, proteasome inhibitors, immunomodulators/antiangiogenic, and immunotherapy with monoclonal antibodies [4]. Despite these advances, MM still remains an incurable cancer and new strategies are urgently needed [3,4].

Here we show that a single miRNA, miR-335, is sufficient to induce apoptosis of MM cells in vitro. We attribute this effect principally to the ability of miR-335 to target the transcription factor *SOX4*, which is overexpressed in many different cancer types [5,8,9,22,23]. In breast cancer, *SOX4* is elevated and induces epithelial–mesenchymal transition (EMT) and metastasis [6,18,24]. Restoring miR-335 content downregulates *SOX4*, modifying the tumor microenvironment and markedly reducing tumor formation [18]. Similar effects have been reported in prostate cancer [10,17] and liver cancer [23]. Loss or gain-of-function studies in acute lymphoblastic leukemia (ALL) cells identified *SOX4* as a critical activator of cell proliferation and survival, and showed that it binds to, and transcriptionally activates, promoters of multiple components of the PI3K/AKT and MAPK signaling pathways [6,21].

In MM *SOX4* regulates transcription of genes involved in cell survival, cell proliferation, cell growth, cell adhesion, migration, and metastasis [8,9,22,23]. Even if it is well known that *SOX4* plays a crucial role in the B cell maturation process, its role in MM remains unclear. In the B cell lineage, *SOX4* expression is high until the pro-B cell and becomes basal in mature B cells. In terminally differentiated plasma cells, the expression increases again (The Human Protein Atlas), suggesting that strategies to deregulate this transcription factor could be used to block tumor differentiation and reprogram cell fate.

Here, we showed an inverse correlation between miR-335 and *SOX4* in MM cells. First, in a model of *SOX4* upregulation via TGFβ-1, since this cytokine is produced in higher levels in MM, by both tumor cells and BMSCs, exerts inhibitory effects on normal B-cell proliferation and Ig secretion [12], and regulates the secretion IL-6 in MM cells [25]. Furthermore, we show that miR-335 down-regulates not only *SOX4* but also other *SOX4*-dependent genes while enhancing AKT expression. Oddly, we found an increase in AKT expression, which we believe to be part of a feed-back mechanism to compensate for the decrease in pAKT [26]. Because *SOX4* downregulation by miR-335 reduced phospho-AKT content it appears as if *SOX4* is also necessary for the maintenance of PTEN/PI3K/AKT activity [16,27,28]. In the PI3K/AKT axis, a crucial role is played by PTEN, which preferentially dephosphorylates phosphoinositide substrates and functions as a tumor suppressor by negatively regulating AKT dependent signaling pathway [21,26]. Oddly, we found low PTEN levels in miR-335 treated cells relative to controls, in agreement with a previous report showing that PTEN is itself a target for miR-335 [27]. This effect apparently overruled PTEN upregulation by AKT dephosphorylation.

An unexpected effect of increasing miR-335 levels in MM cells was a heightened expression of HIF1α. Myeloma cells are known to produce numerous angiogenic regulators including HIF1α and vascular endothelial growth factor (VEGF) [29]. HIF1α plays a crucial role in MM pathogenesis by enhancing the cell’s ability to withstand low oxygen tension [1]. Since we showed that miR-335 induces apoptosis it appears that the two events (downregulation of *SOX4* and activation of HIF1α) are functionally coupled. The reasons why miR-335 transfected into U266B1 or delivered via iEVs increases HIF1α gene transcription and protein expression remain unclear and will need to be addressed in future studies.

## 5. Conclusions

The effects of miR-335 on MM cells targeted using miR-335-laden iEVs opens new perspectives for MM therapy. Co-culture of MM cells with iEVs showed a downregulation of *SOX4* and the induction of apoptosis in MM cells. This is consistent with a study showing that miR-335-laden iEVs markedly inhibit the orthotopic growth of human triple negative breast cancer cells in NSG mice [18]. The present report supports, therefore, the idea that miR-335 delivered either by programmed B cells or by iEVs from programmed B cells can be used to target MM cells as a new form of therapy.

## Supplementary Materials

The following are available online at https://www.mdpi.com/article/10.3390/jpm11121240/s1, Figure S1: Analysis of *SOX4* RNA expression in MM cells compared with Bcells. *SOX4* expression level in MM cell lines evaluated by RT-qPCR using B cells as control. The analysis was the combination of three experiments running each one in triplicate using the threshold.
