# Supplementary material for: miR-335-laden B Cell-Derived Extracellular Vesicles Promote SOX4-Dependent Apoptosis in Human Multiple Myeloma Cells
Source: J Pers Med. 2021 Nov 23;11(12):1240. doi: 10.3390/jpm11121240 (PMC8707697; doi:10.3390/jpm11121240)
Supplement: Supplementary file 1 [file jpm-11-01240-s001.zip › jpm-1452677-supplementary.pdf]

Supplementary Matherial

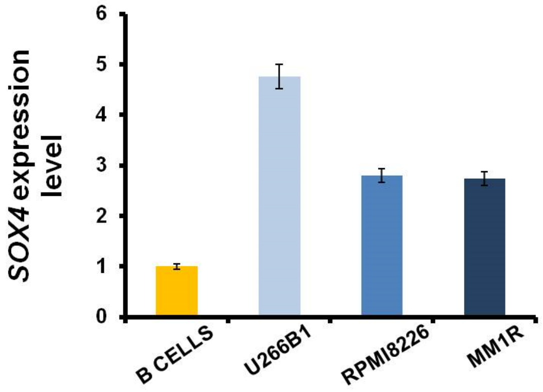

Figure S1. Analysis of *SOX4* RNA expression in MM cells compared with Bcells. SOX4 expression level in MM cell lines evaluated by RT-qPCR using B cells as control. The analysis was the combination of three experiments running each one in triplicate using the threshold.
